# The Design of a Low-Noise CMOS Image Sensor Using a Hybrid Single-Slope Analog-to-Digital Converter

**DOI:** 10.3390/s24248131

**Published:** 2024-12-19

**Authors:** Hyun Seon Choo, Da-Hyeon Youn, Hyunggyu Choi, Gi Yeol Kim, Soo Youn Kim

**Affiliations:** Department of System Semiconductor, Dongguk University, Seoul 04620, Republic of Korea; hs1210@dongguk.edu (H.S.C.); ekgus4020@dgu.ac.kr (D.-H.Y.); hkmymom@dgu.ac.kr (H.C.); powergy@dgu.ac.kr (G.Y.K.)

**Keywords:** CMOS image sensor, correlated double sampling, double data rate, hybrid single-slope ADC, low noise

## Abstract

In this study, we describe a low-noise complementary metal-oxide semiconductor (CMOS) image sensor (CIS) with a 10/11-bit hybrid single-slope analog-to-digital converter (SS-ADC). The proposed hybrid SS-ADC provides a resolution of 11 bits in low-light and 10 bits in high-light. To this end, in the low-light section, the digital-correlated double sampling method using a double data rate structure was used to obtain a noise performance similar to that of the 11-bit SS-ADC under low-light conditions, while maintaining linear in-out characteristics. The CIS with the proposed 10/11-bit hybrid SS-ADC was fabricated using a 110 nm 1-poly 4-metal CIS process. The measurement results showed that dark random noise was reduced by 8% in low light when using the proposed hybrid SS-ADC, compared with the existing 10-bit ADC. Additionally, in the case of high brightness, when using a 10-bit resolution, the dynamic power consumption decreased by approximately 31%, compared to the 11-bit ADC. The total power consumption is 3.9 mW at 15 fps when the analog, pixel, and digital supply voltages are 3.3 V, 3.3 V, and 1.5 V, respectively.

## 1. Introduction

The market for CMOS image sensors (CISs) has been growing steadily, owing to the increasing demand for various applications, such as mobile devices, computer vision, and medical devices [1,2]. Consequently, there has been an increasing interest in basic CIS functionality and Wide Dynamic Range (WDR) CISs, which can capture a broader range of light intensities, and low-noise CISs, which aim to reduce noise under low-light conditions. Various approaches have been proposed to develop WDR and low-noise CISs [3,4,5]. However, in-pixel WDR methods, such as those described in [1,2], suffer from issues related to process incompatibility, leading to problems such as image lag, distortion caused by frame delays and significant vertical fixed-pattern noise (FPN).

Furthermore, applying gamma correction as proposed in [3,4,5] requires additional memory circuitry in image sensors equipped with logarithmic counters. Gamma correction leads to adjustments in low-light regions primarily, which can result in information loss in high-intensity regions following the correction. The gamma correction technique performs correlated double sampling (CDS) at a frequency twice as fast as when a low-light input signal is input than when a high-light input signal is received, resulting in a Signal-to-Noise Ratio (SNR) improvement effect similar to the results of correlated multiple sampling (CMS). CMS is a technique that effectively reduces the standard deviation of low-light random noise by averaging repeated sampling results. When the number of repeated samplings is *N*, there is a noise reduction effect of the square root N [6,7].

**Figure 1:**
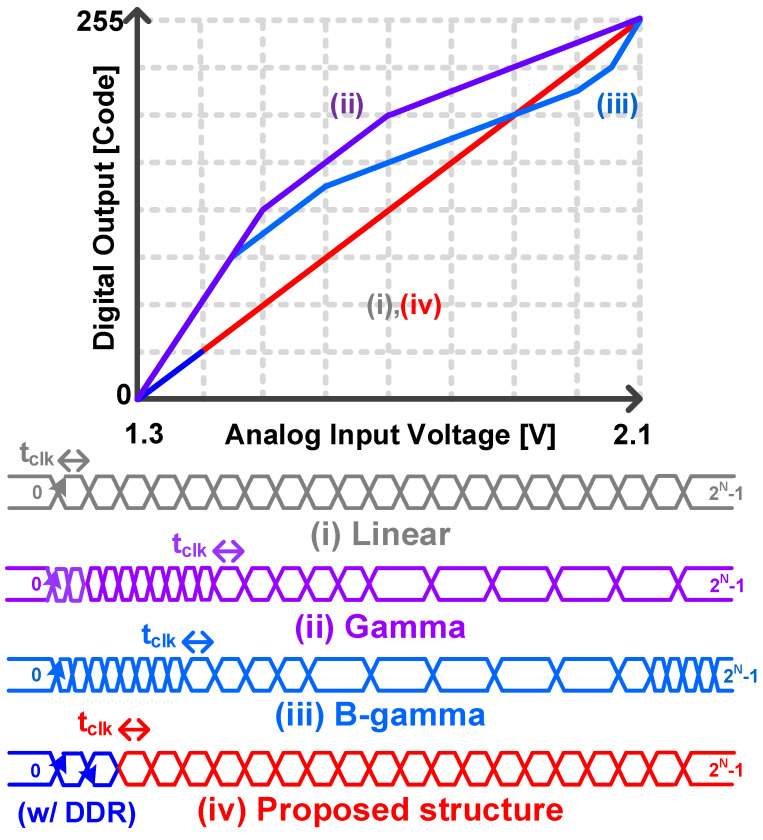
Comparison of the transfer curves and output codes of conventional, gamma-corrected, and B-gamma-corrected CISs and the proposed CIS.

Similarly, in the method described in [3], gamma correction was applied in segments to improve the dynamic range; however, this approach significantly increased the exposure time of the pixels. To address these issues, a bidirectional gamma (B-gamma) correction WDR CIS, which adjusts both low-light and high-intensity regions simultaneously, has been proposed [3]. As shown in Figure 1, gamma and B-gamma show nonlinear input–output characteristics. Therefore, additional computation is required to generate a linear response at the image signal processor (ISP) stage connected to the sensor [8].

In this study, we aimed to implement a low-noise CIS by designing a 10/11-bit hybrid Single-Slope Analog-to-Digital Converter (SS-ADC) that enables a partially-higher-resolution output in low-light conditions, while maintaining an overall linear response. Figure 1 addresses the shortcomings of the aforementioned gamma correction and bidirectional gamma correction methods, which exhibit nonlinear in–out characteristics. The linear response allows for better noise reduction by applying digitally correlated double sampling (D-CDS), which minimizes the error voltages during noise reduction. Additionally, by enhancing the resolution only in low-light regions, rather than across the entire image, the toggling frequency of the counter does not increase for the full counting range, thereby improving the output of meaningful data in the image. To implement the proposed architecture, a double data rate (DDR) structure was applied to the Least Significant Bit (LSB) stage of the column counter, allowing digital codes with an increased 1-bit resolution to be output only in the desired regions. It should be noted that all modes had the same data conversion time when the clock frequencies were adjusted.

**Figure 2:**
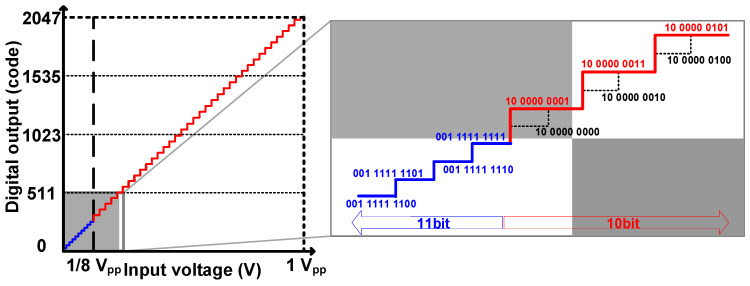
The detailed output response of the proposed 10/11-bit hybrid SS-ADC.

The output characteristics of the proposed approach are shown in Figure 2. In the range of output codes that predominantly appear in dark images, such as those below 1/8 of the full code, the image is output with an 11-bit resolution, where one additional bit is used. For the subsequent code range, the edge-triggered data flip-flop (ETDFF) used for the LSB in DDR is turned off, maintaining the HIGH of the LSB stage.

Furthermore, this implementation results in a lower power consumption, compared to a conventional 11-bit resolution Single-Slope ADC, while maintaining the linearity of the entire output code by producing 10-bit resolution images for regions other than low-light areas. Furthermore, the LSB control block was designed to allow for an adjustable output code region with an 11-bit resolution. The remainder of this paper is organized as follows. In Section 2, we present the design of a low-noise CIS by using the proposed D-CDS method. In Section 3, the layout, simulation, and measurement results are discussed. Finally, the conclusions are presented in Section 4.

## 2. Proposed CIS Structure

**Figure 3:**
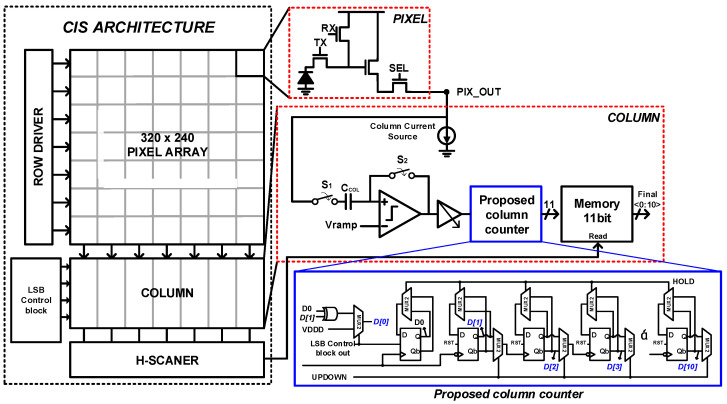
Full block diagram of the proposed CIS.

Figure 3 shows the overall block diagram of the proposed CIS. The pixel uses a 4-transistor Active Pixel Sensor (4-Tr APS) structure, and the memory was implemented using the ETDFF. The proposed column counter structure consists of an LSB counter unit implemented using a DDR and an Up/Down (U/D) counter unit for the subsequent bits. The U/D counter unit comprises two multiplexers (MUXs) that determine the HOLD and Up/Down operations, and one ETDFF. Although the proposed structure operates based on Digital Correlated Double Sampling (D-CDS), Analog Correlated Double Sampling (A-CDS) is also performed during the conversion process to allow the ADC to operate, regardless of the pixel voltage range input.

The operation sequence of the proposed CIS is as follows. The pixel array was controlled by a row driver. The reset voltage of the pixels was down-counted using a column counter. When the reset voltage matches the ramp voltage, the comparator flips its output, causing the counter to stop toggling and to latch the data. Subsequently, the signal voltage, which decreased in proportion to the incident light, was compared to the ramp voltage of the comparator. The column counter up-counts to offset the voltage generated by the same pixel and toggles until the ramp voltage matches the signal voltage.

During the up-counting process, the LSB, D[0], toggles via the DDR LSB unit, outputting an 11-bit code, whereas the LSB_CONT signal from the LSB control block is HIGH. When LSB_CONT becomes LOW, the DDR LSB unit stops toggling, resulting in an output of the 10-bit code. When the ramp signal matches the signal voltage, the column counter output is latched and stops toggling, thereby outputting the effective signal value as a digital code. The counter output is then stored in memory and, subsequently, output through the control by the H-scanner.

### 2.1. A 10/11-Bit Hybrid ADC

**Figure 4:**
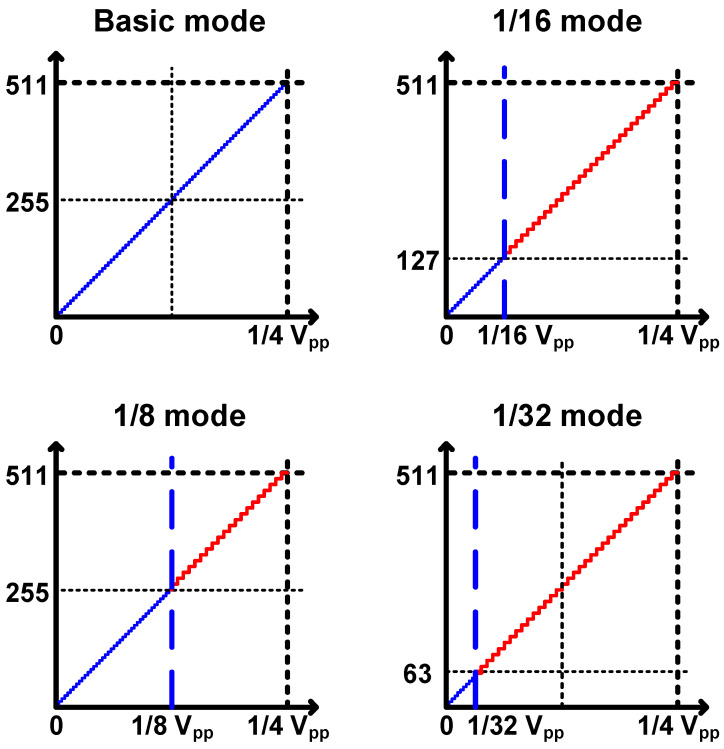
Output code range of 11-bit resolution for each operation mode (blue: DDR readout, red: normal readout, default: 1/32 mode).

A 10/11-bit hybrid ADC was proposed for a low-noise CIS with linear output-response characteristics. In the case of multiple sampling, the noise reduction ratio theoretically corresponds to the square root of *N* for the number of multiples *N* [9]. Because the image output of the Log-exponential Complex Counter (LCC) was corrected by adjusting the digital gain, the images were compared using the Modified Absolute Mean Brightness Error (M-AMBE) [10]. Because the colors of the color blocks inside the Macbeth ColorChecker, a color chart, are constant, each color of the blocks is converted to a standard RGB (sRGB), expressed as R, G, and B values, and then converted to grayscale for actual measurements using the values as a standard. The M-AMBEs of each image were compared. As a result of the comparison, the M-AMBE improvement in images taken at 20 lx, a low-light area (=1/16 or less of the total output code), was found to be up to approximately 20% [10]. In the case of the proposed SS-ADC, multiple sampling areas were designated using a counter, and the ADC had ADC characteristics of 11 bits under low illumination and 10 bits under high illumination. Therefore, we analyzed the noise reduction effect of the final output by adjusting the transition point from 10-bit to 11-bit to 1/8, 1/16, and 1/32 of the total output code, as shown in Figure 4.

### 2.2. Conventional U/D Counter

**Figure 5:**
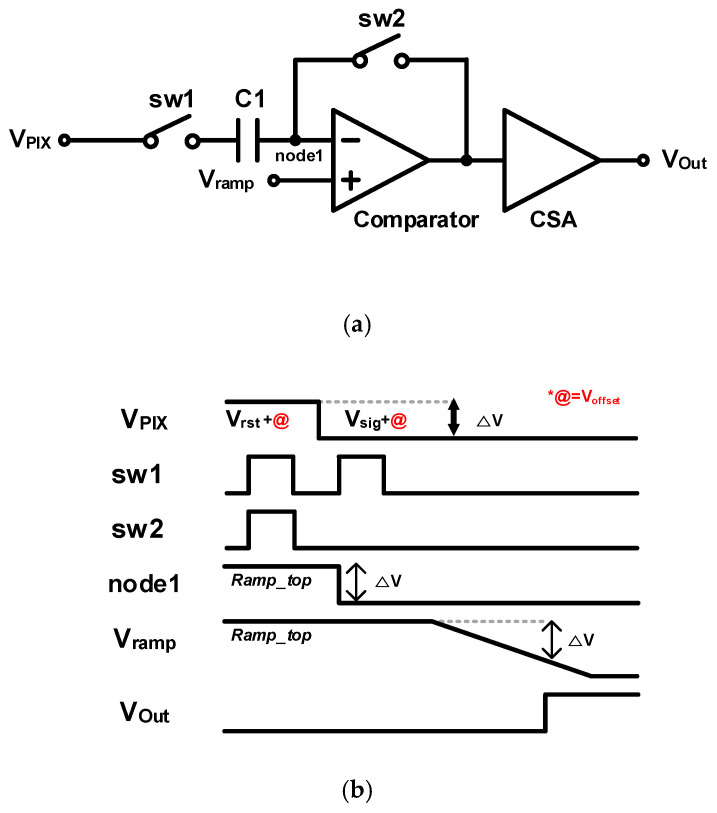
(**a**) Block diagram and (**b**) a timing diagram of the comparator.

The proposed structure operates based on the D-CDS. However, for the ADC to operate, regardless of the range of the pixel voltage input to the ADC, A-CDS is also performed during the conversion process using a DC blocking capacitor, as shown in Figure 5a. Figure 5b illustrates the timing diagram of the A-CDS, which performs voltage distribution according to the law of charge conservation.

**Figure 6:**
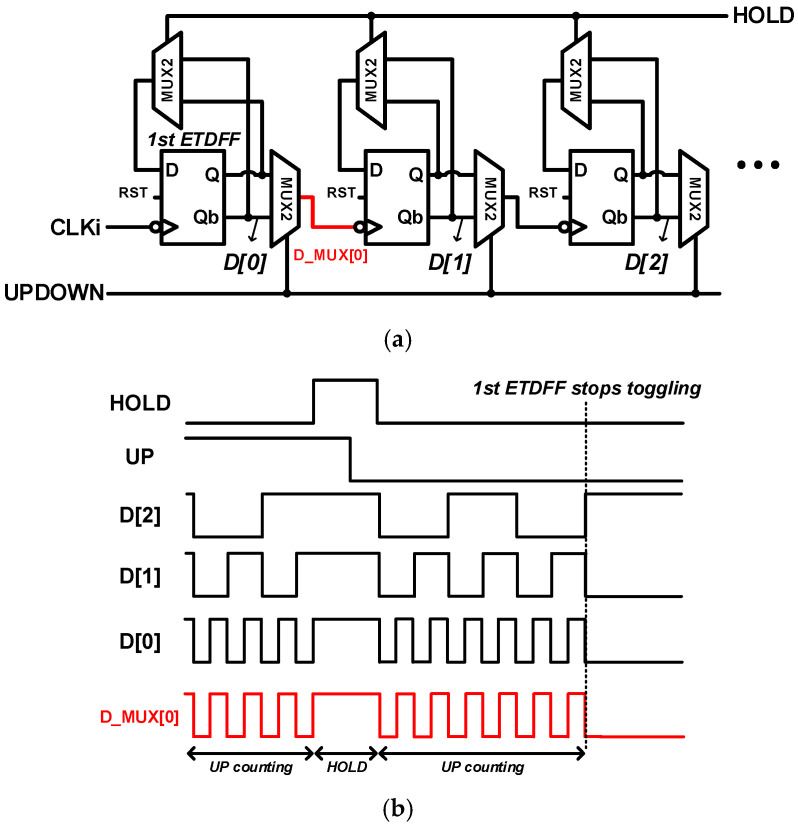
(**a**) Conventional U/D counter structure and (**b**) issue.

Figure 6 shows the block diagram of a conventional U/D counter. In the case of a method that applies bidirectional gamma correction by adjusting the digital gain using LCC, when compared to a conventional SS-ADC structure, the output code at a low-illuminance (approximately 0–255 out of 2047) range and Signal-to-Noise Ratio (SNR) were improved [3]. However, in the case of high illuminance, the noise improvement effect is negligible because photon shot noise is dominant.

In addition, when applying gamma correction and bidirectional gamma correction, additional calculations are inevitable in the image signal processor (ISP) connected to the back of the image sensor, owing to the nonlinear input–output response characteristics. Accordingly, the image and noise were improved only by increasing the digital gain in the low-light area. However, a structure that maintains an overall linearity by applying a normal gain (×1) in the high-light area is still required. However, as mentioned earlier, additional bits of resolution output only in low-light conditions cannot be implemented with the existing U/D counter structure. When the LSB stage ceases to operate after switching off in low-light areas, the operation of the entire counter stops because of the structure of the counter, in which the output of the previous stage is used as the input for the next stage. The simulation results are shown in Figure 6, which shows the stopping of the toggling of the first ETDFF. To solve this issue, the proposed 10/11-bit hybrid SS-ADC uses a DDR structure to separate the rest of the LSB stage from the other counter stages.

### 2.3. The Proposed Counter Using a Double Data Rate for the 10/11-Bit Hybrid ADC

**Figure 7:**
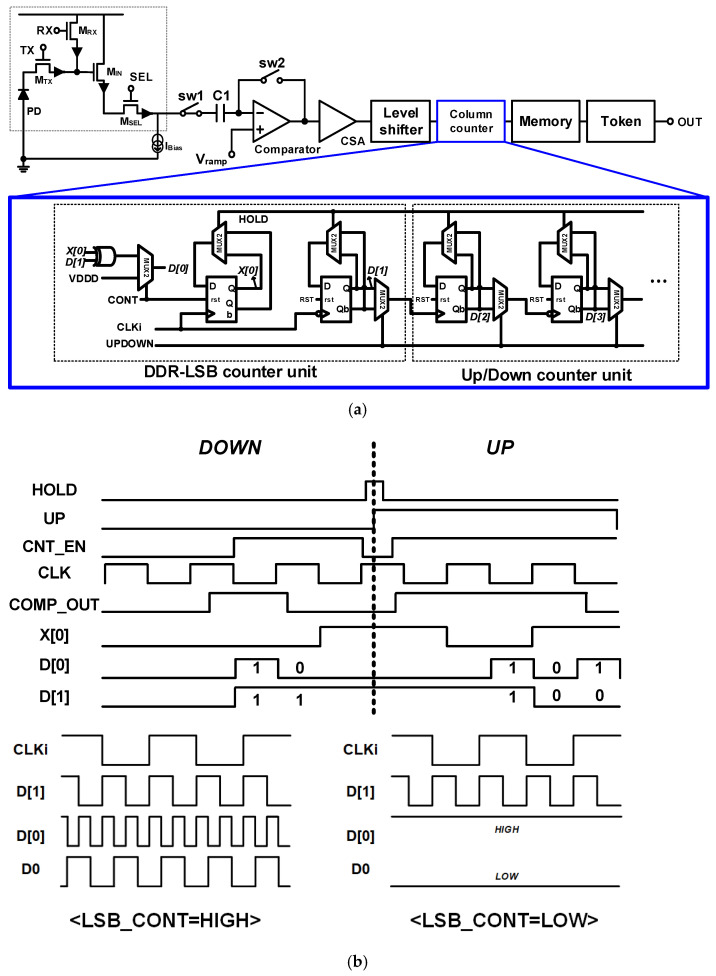
(**a**) One-column structure of the proposed CIS and (**b**) DDR-LSB block output according to the LSB_CONT signal.

Figure 7 shows the proposed one-column CIS structure and the operation of the proposed U/D counter with DDR to obtain a 10/11-bit hybrid ADC operation. Compared to the single data rate (SDR), the counter structure with a DDR enables twice the data transmission with the same clock signal. The DDR structure was implemented inside the U/D counter using a negative ETDFF, positive ETDFF, and XOR.

**Figure 8:**
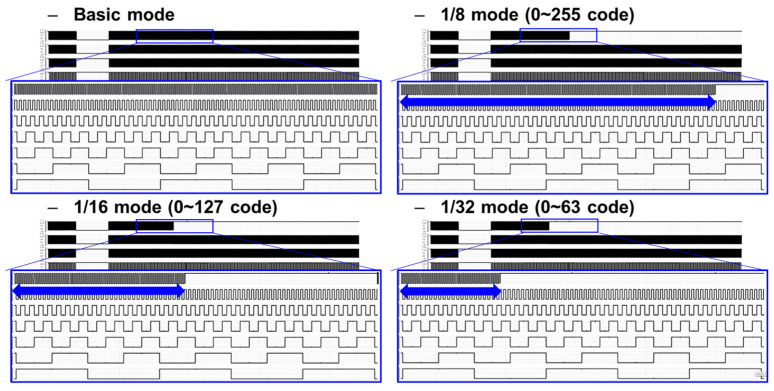
Simulation results for each mode of the proposed column counter.

Figure 8 shows the output for each mode of the LSB control block. The CIS proposed to control the change point from 11-bit to 10-bit, which can operate in four modes using the CNTL [0:1] of the LSB control block. It is possible to check the change in noise by adjusting the mode-change point. The simulation results confirm the mode-change point that outputs an additional 1-bit resolution for each mode change, as shown in Figure 8. Design simulations and analyses were performed for normal operating conditions with 3.3 and 1.5 V of analog and digital supply voltages, 10 MHz of main clock frequency (=data clock frequency in this work for 15 FPS with 0.27 ms of 1 horizontal time, 1H-time), and an operating temperature of 25 °C.

## 3. Simulation and Experimental Results

### 3.1. Monte Carlo Simulation Results

**Figure 9:**
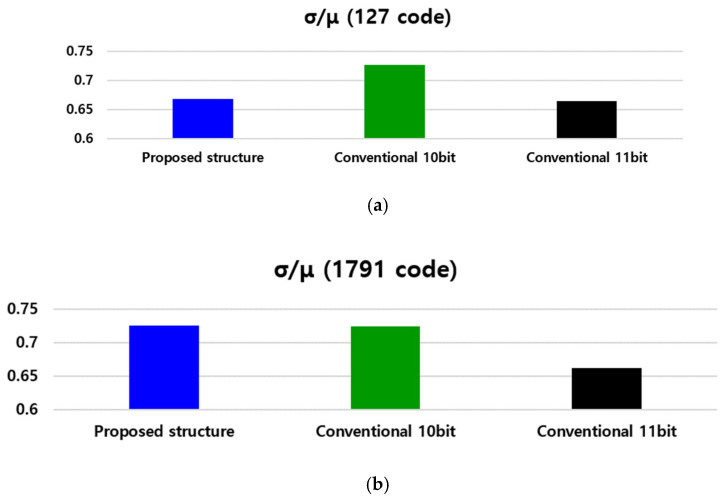
Comparison of Monte Carlo simulation results with different resolutions: (**a**) under low-illumination and (**b**) high-illumination conditions.

For the full-chip transient simulation results, we assumed that the range of the reset voltage was 2.3~2.1 V, that of the ramp voltage was 2.3~1.3 V, and that of the voltage of one LSB code was 0.39 mV. When the actual effective pinning voltage was set to approximately 685 mV, the simulation results confirmed that the same 1755 code was output by counting the area where the additional 11 bits were outputted. Figure 9 shows the results of a Monte Carlo simulation of the proposed column counter structure with 1000 Monte Carlo points. In the case of the SS-ADC structure, the counter operates at a constant time; therefore, it is possible to calculate the time at which a specific code is output. Therefore, based on the point at which the 127 code, which is a code output in a low-illuminance area, and the 1791 code, which is a code output in a high-illuminance area, are output, there are three structures (10/11-bit hybrid ADC, 10-bit ADC, and 11-bit ADC). The σ/μ (standard deviation/center of a collection of numbers) of the voltage at the time point was compared. For the 127 code in the low-light area, the proposed structure shows σ/μ results that are almost similar to those of a typical 11-bit SS-ADC. However, it was confirmed that σ/μ decreased by approximately 8%, compared with a typical 10-bit SS-ADC. For the 1791 code output in the high-intensity area, the noise performance is similar to that of the proposed structure and a typical 10-bit structure. By examining the Monte Carlo simulation results, it was statistically confirmed that the proposed structure exhibits a noise performance similar to that of a typical 11-bit structure under low light.

### 3.2. Measurement Results

**Figure 10:**
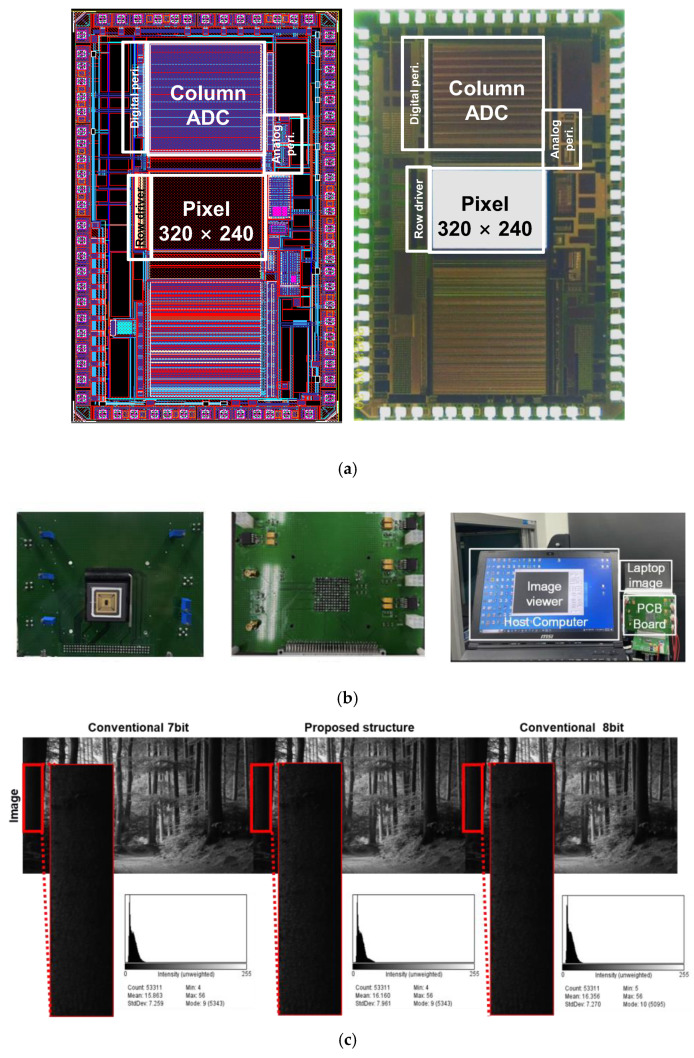
(**a**) Layout and chip microphotograph, (**b**) a proposed CIS on the printed circuit board (front/back sides) and test environment, and (**c**) comparison of images and histograms with the 7-bit, 7/8-bit hybrid, and 8-bit CISs.

The prototype chip of the proposed CIS was fabricated using a 0.11 μm CMOS process. Figure 10a illustrates the layout and photograph of the fabricated prototype CIS chip, which occupies an area of 2.55 mm × 5.24 mm. The photodetector is a non-shared 4T-APS structure with an area of 3.25 μm × 3.25 μm. When the supply voltage of APS is 3.3 V, the pinning voltage is about 2.0 V. Control signals for operating the image sensor were generated using an FPGA board, XEM3050 (Xilinx Spartan-3 FPGA Integration Module, Santa Clara, CA, USA) connected to a computer. These control signals were implemented in Verilog using the Integrated Scripting Environment (ISE) 14.7 software and applied to the designated pins of the image sensor on the printed circuit board, as shown in Figure 10b. After completing Verilog coding, the output signals from the sensor were sequentially read and verified using an image viewer on a computer. The FPGA was driven by a USB interface using Xilinx Opal Kelly board, and the final image output was assessed using an image viewer.

Figure 10c shows an example of an image captured by the proposed CIS. Owing to the resolution limitations of the image-viewer software, the operation results were confirmed for the 7/8-bit hybrid ADC instead of the 10/11-bit configuration. The measurement environment was as follows: analog and digital supply voltages of 3.3/1.5 V, main clock frequency of 10 MHz.

As a result of enlarging the low-light code area, it was confirmed that the proposed hybrid structure (center images) increased the type of code expressed, compared with the existing 7-bit counter, thereby increasing the standard deviation of the code in the low-light area. These results indicate that the diversity of the code expressed has increased, and that visibility has improved. The measured SNR of the 1/32 mode increased by 23% in low-light areas where SNR was limited by read noise, as shown in Table 1. Conversely, there is little noise reduction in high-light areas where SNR is limited by shot noise. In addition, the SNR results of the 1/32 mode and 1/8 mode were almost similar.

**Figure 11:**
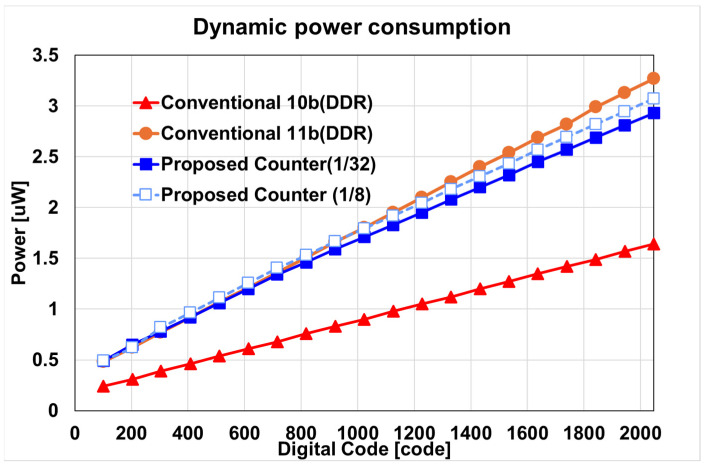
Comparison of dynamic power consumption of a one-column counter with different resolutions of ADC and the proposed structure with 1/8 and 1/32 modes.

In Figure 11, the power consumptions of the proposed column counter structure and a typical counter using DDR are compared. It can be observed that the proposed structure with a 1/32 mode reduces the power consumption by up to 31.2%, compared to a general 11-bit counter with DDR applied. Additionally, when comparing the 1/8 and 1/32 modes, in the case of the 1/8 mode, the number of high-speed operations is four times greater, so the power reduction effect is lower than that of the 1/32 mode. Comparing the output image and power consumption, it can be observed that the proposed structure can achieve image improvement while consuming less power than a typical counter structure under low light.

Table 2 provides a performance comparison of low-noise CISs. Compared with the gamma correction CIS in [3], the proposed design achieved a reduction of up to 9% in dark random noise under low-light conditions through the implementation of dual sampling. In addition, the proposed CIS demonstrated a reduction in dark random noise of up to 35%, compared with the pseudo-multiple sampling-based approach in [11], which is known for its low dark random noise but high power consumption. Furthermore, at low illumination levels, the proposed CIS achieved a 40% improvement in the figure of merit (FoM) compared to [3].

## 4. Conclusions

In this paper, we present a low-noise CMOS image sensor (CIS) featuring a 10/11-bit hybrid single-slope analog-to-digital converter (SS-ADC) to optimize its performance under varying lighting conditions. The proposed hybrid SS-ADC achieves an 11-bit resolution under low-light conditions and a 10-bit resolution in high-light scenarios. By employing a digital-correlated double sampling method with a double data rate structure in the low-light section, the CIS demonstrated a noise performance comparable to that of an 11-bit SS-ADC in low-light environments. The measurement results show that the CIS reduced the noise level by approximately 31% when operating at a 10-bit resolution, compared to an 11-bit ADC. These results demonstrate the ability of the proposed hybrid SS-ADC to achieve an optimal balance between noise performance and resolution across different illumination levels, thereby offering a practical solution for low-power, high-performance image-sensing applications.

## Figures and Tables

**Table 1:** Measured output [mV], noise [mV] and SNR [dB] with different illuminations [Lux].

Illumination	Output	10bit SS-ADC SNR	Proposed Counter (1/32) SNR
100	39.50	20.22	24.88
340	95.39	21.38	25.89
900	275.39	28.67	29.50

**Table 2:** Comparison of the performance characteristics of the proposed CIS with other works.

	[3]	[10]	This Work
Technology (nm)	110	90	110
Image Resolution	640 × 480	960 × 720	320 × 240
Pixel Size (μm^2^)	3.2 × 3.2	1.4 × 1.4	3.25 × 3.25
ADC Column Pitch (μm)	3.2	5.6	3.25
ADC Resolution (bit)	8	10	11
FPS (frames/s)	15	15	15 (75 at max.)
Random Noise (V_RMS_)	334 μ	472 μ	304 μ
Total Power (mW)	6	280	3.9
FoM (V_RMS_·W/FPS·# of Pixels)	435 μ	542.8 μ	261 μ

## Data Availability

The datasets generated in this study are available from the corresponding author upon reasonable request.
